# Cosmetic Revision Surgeries after Transfeminine Vaginoplasty

**DOI:** 10.1007/s00266-022-03029-9

**Published:** 2022-08-24

**Authors:** Ivan Mañero, Anna I. Arno, Roberto Herrero, Trinidad Labanca

**Affiliations:** 1 “Dr. Ivan Mañero” Institute of Plastic Surgery (IMCLINIC), Carrer de Victor Hugo 24, Sant Cugat del Vallès, 08174 Barcelona, Spain; 2 “University of Barcelona” (UB), Barcelona, Spain; 3 “Universitat Autònoma de Barcelona” (UAB), Barcelona, Spain; 4 “Hospital Dr. Bernardo Houssay”, Pres. Hipólito Yrigoyen 1757, Vicente López, Buenos Aires, Argentina

**Keywords:** Vaginoplasty, Cosmetic revision surgery, Transfeminine gender-affirming surgery

## Abstract

**Background:**

Vaginoplasty is the most frequent genital gender-affirming surgery. Although both functional and aesthetic outcomes after transfeminine vaginoplasty have improved over the years, cosmetic revision surgeries demand after transfeminine vaginoplasty appears to be increasing and requires updated knowledge.

**Methods:**

All patients who underwent vulvar cosmetic revision surgeries at our institution following transfeminine vaginoplasty from January 2014 to April  2022 were studied. The prevalence, topography and surgical techniques of cosmetic revision surgeries after transfeminine genital gender-affirming surgery were examined using clinical charts review and statistical analysis.

**Results:**

During the study period, 354 patients underwent gender-affirming vaginoplasty at our single institution (212 penile inversion vaginoplasty, 122 colovaginoplasty and 20 penile inversion vaginoplasty with scrotal skin graft patients). Forty out of these 354 patients (11.29%) required cosmetic revision surgery after transfeminine vaginoplasty; additionally, 44 patients with vaginoplasty performed at other centres also underwent vulvar cosmetic revision surgery at our clinic during the study period. From all performed cosmetic revision surgeries, most of them (31.42%) were labia corrections, followed by clitoris (23.26%) repair surgeries. Mons Venus (10.20%), urethral meatus (9.38%), spongiosus tissue remnants (8.57%) and introitus (6.53%) revisions followed in frequency. Corrections of peri-inguinal scars (5.30%), anterior commissure (2.84%) and inferior fourchette (2.42%) were less prevalent. No differences were found among the different studied vaginoplasty techniques regarding cosmetic revision surgery prevalence or topography following transfeminine vaginoplasty (*p* < 0.05).

**Conclusions:**

Cosmetic revision surgeries after transfeminine vaginoplasty are frequent. In our large and long-term cohort study, labiaplasty followed by clitoroplasty were found as the most required cosmetic revision surgical procedures. Further multicentre, prospective and controlled studies are necessary to improve cosmetic outcomes and scientific evidence after transfeminine vaginoplasty.

**Level of evidence IV:**

This journal requires that authors assign a level of evidence to each article. For a full description of these Evidence-Based Medicine ratings, please refer to the Table of Contents or the online Instructions to Authors www.springer.com/00266.

## Introduction

Functional and aesthetic outcomes after vaginoplasty as gender-affirming surgery have enormously improved during most recent years, due to the innovative surgical changes introduced by expert surgeons and to a growing demand and interest on this type of surgery [1].

In this regard, vaginoplasty has transitioned from being an almost completely reconstructive surgery performed on several surgical stages, mainly with a functional focus centred on the vagina—not taking first into account the aesthetic appearance of the vulvar region—to a most complete and refined surgery on a single surgical stage [2]. Nowadays, besides creating a functional neovaginal tunnel in those patients desiring sexual penetration, a high-quality vulvoplasty has been achieved.

However, it appears that there is a popular increasing demand of genital cosmetic surgery, not only in biological cis-women [3], but also in transgender women. With a described prevalence of 20–50% [4], cosmetic revision surgeries after vaginoplasty include scar revision, lipofilling, removal of excess skin [5] and/or mucosa, mons pubis plasty [6] and urinary meatus refinement [7], among others. Preoperative patient education is necessary to counsel transfeminine patients on realistic expectations and outcomes [4]*.*

There is few quality scientific evidence regarding functional complications after transfeminine gender-affirming surgery, but aesthetic complications have been even less studied in the literature. Therefore, further research on aesthetic revision surgeries after transfeminine vaginoplasty is mandatory to shed new light on this unexplored issue. Having more information on prevalence and topography of aesthetic revision surgeries after transfeminine vaginoplasty may help to prevent further cosmetic revision surgeries following the primary vaginoplasty procedure. On the other hand, knowing in more detail the aesthetic revision surgery techniques specifically indicated and usually successful in this population may help to progress the transgender surgical field.

The main aim of this observational study is to analyse the prevalence and topography—and describe our up-to-date surgical techniques—of cosmetic revision surgeries performed at our specialized plastic surgery and gender-affirming surgery clinic (IMCLINIC) following transfeminine vaginoplasty (underwent at our or other centres), with large population size and long-term study period. Secondary aims are to examine if there are any differences in cosmetic revision surgeries among the two vaginoplasty  provenance groups and among the different studied transfeminine vaginoplasty types.

## Materials and Methods

In this retrospective observational cohort study, all patients who underwent gender-affirming vaginoplasty at IMCLINIC clinic from January 2014 to April 2022 were included. Vaginoplasty surgery type was recorded as follows: penile inversion vaginoplasty (PIV), colovaginoplasty and penile inversion vaginoplasty with scrotal skin graft (PIV + graft). The prevalence of cosmetic revision surgery following gender-affirming vaginoplasty at our clinic was analysed first. Patients with transfeminine vaginoplasty performed at other centres who underwent vulvar cosmetic revision surgery at our institution during the same time period were also examined. Therefore, all vulvar cosmetic revision surgeries performed at our clinic following transfeminine vaginoplasty of any provenance were studied. All patients met the standards of care set forth by the WPATH and gave informed consent to participate in this research study. This research followed the Declaration of Helsinki principles and was granted Ethics Committee Approval from our affiliated institution, Universitat Autònoma de Barcelona (UAB), with the reference number CEEAH 5689.

The prevalence, topography, and surgical techniques of aesthetic revision surgeries performed by the senior surgeon at IMCLINIC following transfeminine genital-affirming surgery at our clinic or not were examined using clinical charts revision and database statistical analysis. Our technical surgical algorithm of cosmetic revision surgeries following vaginoplasty is described. Differences in cosmetic revision surgery prevalence and topography among different types of transfeminine vaginoplasty were analysed.

We used the SPSS programme, version 2.0, for database and statistical analysis. The median and percentile 25/75 were used to describe quantitative variables. The difference between the qualitative variables was compared using the Chi2test or Fisher test. The quantitative variables were analysed using a nonparametric test (Wilcoxon for paired samples). All statistical tests were two-tailed, with a significant *p* value of less than 0.05.

### Surgical Design/Technique

As per our approach, every vulvar aesthetic revision surgery in transfeminine patients following vaginoplasty is carefully planned and tailored-designed specifically for each patient. The main preoperative analysis focuses on the anatomic structures involved to be repaired and the role they interplay in association with the other satellite perineal structures. Clitoris, Venus Mons or Mons pubis, labia minora, labia majora, vaginal introitus, urinary meatus, postoperative perineal scars, inferior fourchette and anterior labial commissure, as well as spongiosus tissue remnants (if present), are carefully examined.

#### Clitoris

**Fig. 1:**
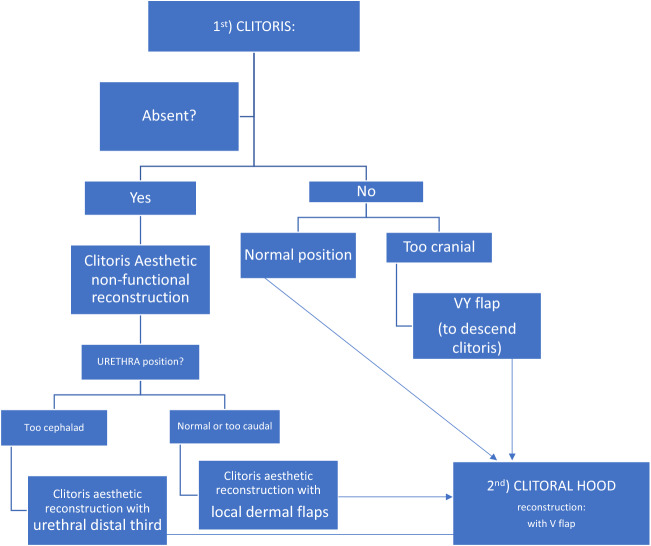
Basic algorithm about clitoris preoperative analysis

**Fig. 2:**
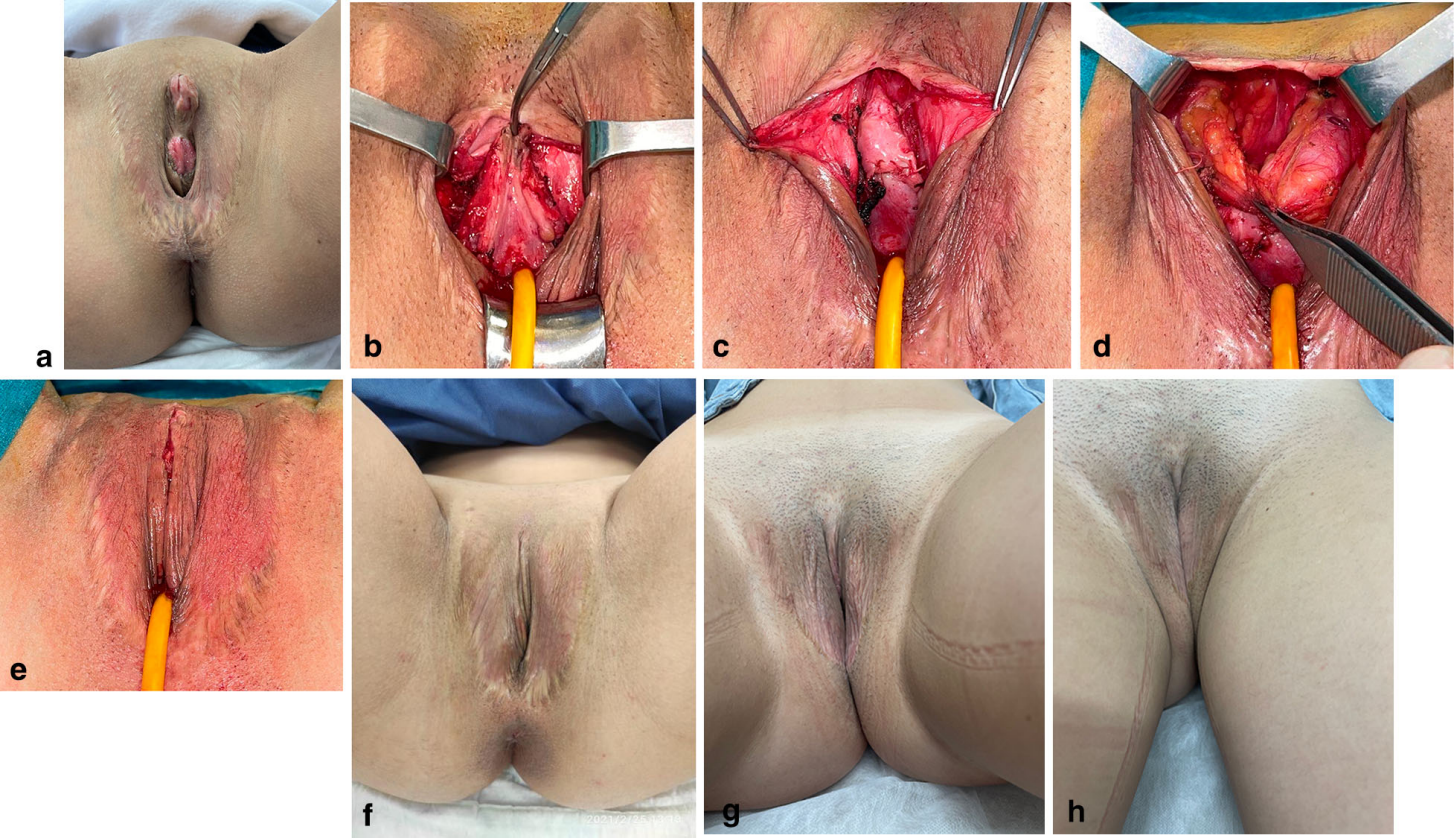
**a–e** Complex Clinical case # 1: Complex Aesthetic multiple vulvar defect after transfeminine vaginoplasty (performed at another centre): **a** Case Presentation: (1) Clitoris abnormalities (being too large and too cranially located, with no clitoral hood), (2) unaesthetic scars, (3) labia minora absence and labia majora defect, (4) bifid Venus mons, (5) urethra malposition (too cephalad), (6) bulbospongiosus tissue remanence, (6) vulvar vestibule, introital and inferior fourchette defects. **b** Cosmetic revision surgery main steps: Step 1. Clitoris and urethra are descended. Urethra is previously spatulated to reconstruct a reddish neovaginal introitus. **c** Cosmetic revision surgery: Step 2. Urethra descend is completed and urethra is shown in the midline. Donor tissue is obtained on both sides after urethra spatulation to reconstruct labia minora. **d** Cosmetic revision surgery: Step 3. Correction of a bifid Venus Mons, and labia majora: Venus Mons bilateral fat is attached at the midline. Labia majora fat is redistributed. **e** Final immediate postoperative result: Vulva appears aesthetically more pleasant and similar to a biological cis-female vulva. **f** Mid-term (1-year) postoperative result: Some cosmetic defects still can be seen, such as labia majora lack of volume and unaesthetic scars, which required a second cosmetic revision. **g**, **h** Long-term (2-year) postoperative result: Two postoperative years since first revision surgery; six months following secondary cosmetic revision surgery

Regarding clitoris cosmetic revision surgery preoperative analysis and planning (Fig. 1), the following facts are evaluated: clitoris existence or not, size and anatomical location, as well as clitoral hood coverage availability. At our plastic surgery and gender-affirming surgery specialized centre, we sometimes encounter patients who underwent vaginoplasty at other clinics with a myriad of other techniques than our usual standardized vaginoplasty approach; in some of these patients, clitoris absence is frequent and, when clitoris is present, clitoromegaly, absence of clitoral hood and/or clitoral malposition is also a common finding (Fig. 2), being the clitoris often located too cranially from the vaginal introitus.

To surgically repair these defects, cosmetic non-functional reconstruction of the clitoris is indicated when this anatomical structure is absent, using local flap techniques. When clitoris is too large, clitoris reduction surgery is offered. When clitoris is too cranially malpositioned, a descending VY local flap elevated with careful clitoral vasculonervous pedicle dissection to prevent any undesired iatrogenic lesions is suggested.

Clitoral hood reconstruction is complex, as there is usually lack of local neighbouring donor tissue. If available, an anterior labial commissuroplasty is recommended, making two oblique incisions, one per side, superolaterally to the clitoral midline, obtaining two triangles building a “dome”. We position the new clitoral hood upper border at the dome vertex, whereas the remaining clitoral hood is left lying at both dome sides, protecting the delicate clitoris anatomical structure.

#### Mons Pubis

In many patients, some months after vaginoplasty, a lateral dislocation or luxation of Venus Mons fatty tissue can be shown, obtaining a “bifid” Venus Mons. The rationale of this lateral fat displacement may reside on two facts: midline location of the maximum tension point and “memory” of the presence of the penile suspensory ligament.

**Fig. 3:**
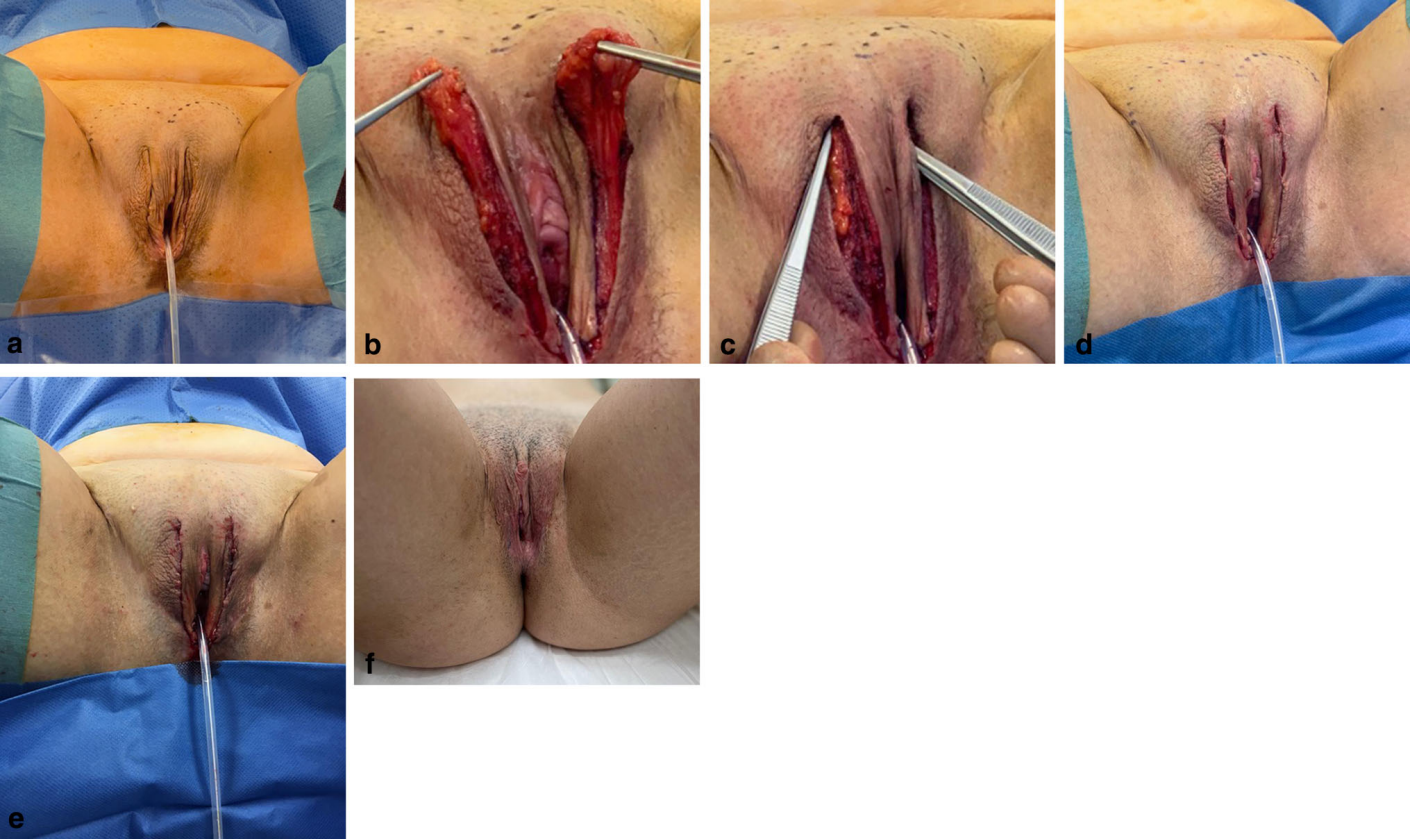
**a**–**f** Cosmetic revision surgery after transfeminine vaginoplasty to correct a bifid Mons Venus: **a** Preoperative design. **b** Dissection and elevation of local fat flaps. **c** Bilateral fat flap transposition, with separate midline and interflap attachments. **d** Direct closure. **e** Immediate postoperative result. **f** Long-term (1-year) postoperative result

The aesthetic revision surgery to repair this bifid Mons pubis consists on a medial transposition of the two local pedicled fat flaps that are laterally displaced, attaching them at the midline independently and also suturing them with each other (Fig. 3). An additional attachment to the pubic symphysis is also recommended to assure more stable and long-lasting results.

#### Labia Minora (Fig. 2c)

Labia minora recreation techniques are scarcely standardized; they are considered complex and very surgeon-dependent. As donor tissue, our approach is to use the remaining tissue following urethra spatulation. In transfeminine patients that undergo a colovaginoplasty as a secondary or salvage vaginoplasty procedure and need labia minora recreation, the previous penile introital skin may also be used.

#### Labia Majora

The labia majora aesthetic defects more frequently seen following vaginoplasty include asymmetry because of size/volume differences (mainly due to localized subjacent fat excess or defect) and skin redundancy.

**Fig. 4:**
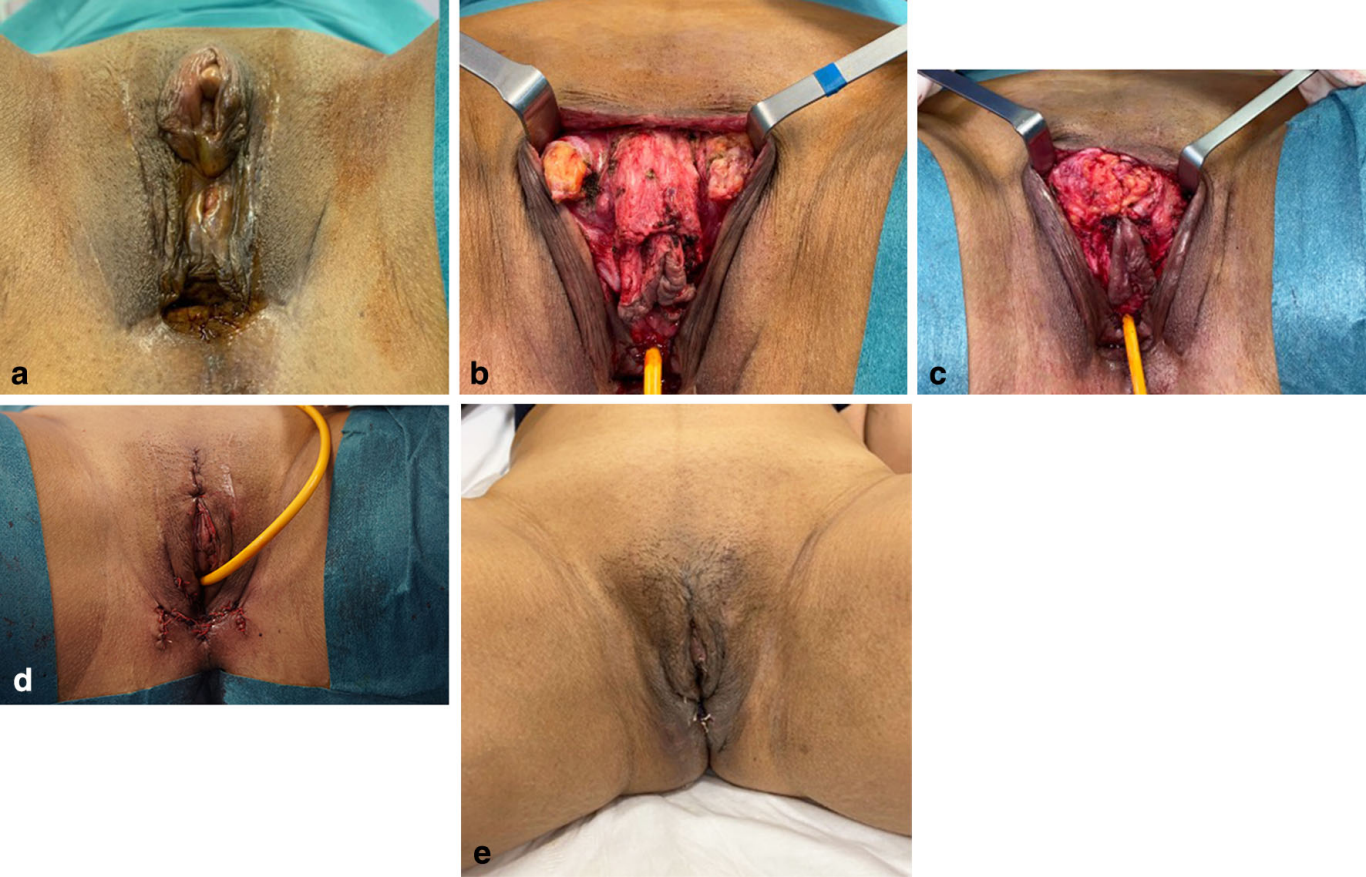
Complex Clinical Case #2 of Cosmetic Revision Surgery after Transfeminine Vaginoplasty: **a** Complex cosmetic multiple vulvar defect after transfeminine vaginoplasty, performed at another centre. **b**, **c** Labia majora cranial bilateral fatty defect presentation and correction, with concomitant other multiple vestibule and vulvar defects. **d**, **e** 1 week postoperative result: Observe that Z-plasties have been made to correct a wide inferior fourchette

Some months after vaginoplasty surgery, when oedema subsides, excess of labia majora skin may be usually encountered, leading to the appearance of wrinkles or folds that reminiscence of the scrotalized origin of the labia majora. Excising this excess of scrotalized skin of the labia majora tends to solve this problem and helps to restore a smooth and youthful appearance to the labia majora in the transfeminine patient. In a similar manner, the cephalic part of the labia majora tends to show an excess of fatty tissue that should be corrected by caudally transposing a local advancement fat flap, attached all along the labia until the inferior fourchette to prevent further relapse (Fig. 4a–c). Another option is to perform a “filling plasty” using local gluteal fat advancement flaps or a lipofilling surgery. On any case, the main goal is to fill the distal third of the labia majora. As per our approach, lipofilling donor area is usually the abdominal region and 15–30 ml of autologous fat is usually grafted per labia. However, it should be noted that fat grafts in the genital region do not tend to take easily; therefore, lipofilling alone is not considered a satisfactory long-term single solution, as per our experience.

#### Vaginal Introitus

Some neovaginas may have an opened introitus at its more caudal margin, which may require direct closure and attachment to the inferior fourchette. In those cases where the introitus has a poor natural appearance mostly due to dyschromia resulting from heterogenicity of satellite tissues in non-intestinal vaginoplasty techniques, our recommendation—ideally during the same surgical step as the primary vaginoplasty—is to spatulate the urethra and use part of its mucosa for introitus reconstruction. By doing so, the neovaginal introitus appears to have a more reddish and natural look, resembling the introitus of a biological cis-female.

**Fig. 5:**
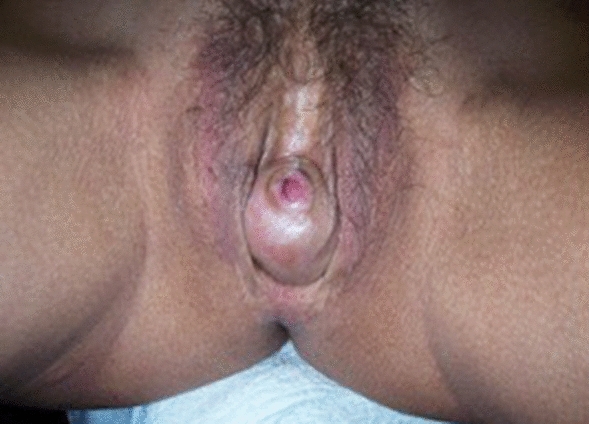
Spongiosus body and bulb remanence: Vulvar defect due to spongiosus body and bulb remanence, which is shown ingurgitated during sexual arousal

On the other hand, one of the aesthetic defects that may affect the neovaginal introitus in transfeminine patients after vaginoplasty may also be linked to a key functional sexual defect: the presence of spongiosus body and bulb remnants. The remanence of spongiosus tissue tends to manifest clinically during sexual arousal, in form of a clear ingurgitation and tension oedema in the neovaginal vestibule, partially or even completely occluding the introitus, disturbing or even not allowing sexual penetration at all (Fig. 5). To solve this concern, ideally during the same surgical step as the initial vaginoplasty surgery, all spongiosus bulbous tissue should be resected; bulbar arteries, ligated; and urethra, spatulated.

#### Urethral Meatus (Figs. 2, 6)

**Fig. 6:**
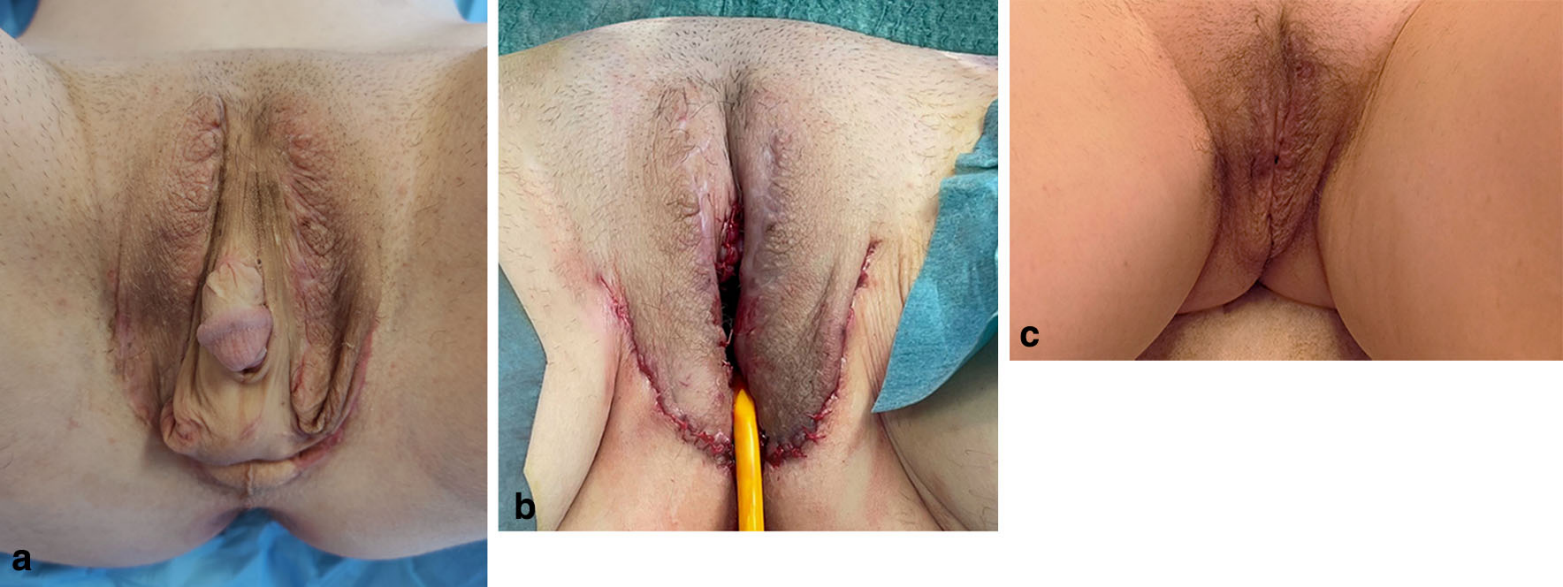
Complex Clinical Case #3 of Cosmetic Revision Surgery after Transfeminine Vaginoplasty: **a** Complex cosmetic multiple vulvar defect after transfeminine vaginoplasty, performed at another centre. **b** Immediate postoperative result (showing clitoris, labia, introitus and urethra cosmetic revision surgery outcomes). **c** Long-term (9 month) postoperative result

In some cases, the urinary meatus and the urethra have an abnormal cephalic position. In these cases, urethra is opened and spatulated as the pages of a book, in order to descend the urethra and position the urinary meatus in a more physiologic anatomic location. Urethra is left lying horizontally to the symphysis. The remaining urethral mucosa is used to line and reconstruct the introitus, as previously described.

#### Scars Satellite to Both Inguinal Folds

Scars at the lateral margins of the labia majora, satellite to both inguinal folds, tend to widen easily following vaginoplasty and they may require aesthetic revision genital surgery, usually with scar removal and a new direct closure.

#### Inferior Fourchette

The inferior fourchette may suffer two main problems: (1) marsupialization and/or (2) lack of volume, leading to a closed or opened inferior fourchette, respectively.

In marsupialization cases, our approach consists on making an incision at the midline for tissue release, leaving two oblique lateral incisions resembling a “V”. It is also important to bring cutaneous, fat and muscular tissue—but mainly by means of a fat flap—to the inferior fourchette, in order to give tension and keep it closed only at its distal margin.

In those patients with a too wide inferior fourchette, it is recommended to perform a surgical reconstruction with a local fat transposition flap. If direct closure with resorbable suture is performed, it should be kept in mind that it may increase the risk of future scar contractures in this area, which may even affect and occlude the vestibule and the neovaginal introitus. In these cases, the solution consists on making a episiotomy with optional additional Z-plasties (Fig. 4d, e).

#### Anterior Labial Commissure

In those cases where the anterior labial commissure appears too wide, correction by a local fat transposition flap is usually sufficient. It is not recommended to perform only direct closure of the commissure at the midline by suture, as this may enhance the formation of undesired and painful scar contractions, especially while rubbing during sexual intercourse.

## Results

From January 2014 to April 2022, 354 patients underwent transfeminine vaginoplasty at our institution, divided in 112 PIV, 122 colovaginoplasties and 20 PIV + graft patients. Forty out of these 354 patients (11.29%) required vulvar cosmetic revision surgeries after transfeminine vaginoplasty. Additionally, 44 patients with vaginoplasty performed at other centres (40 PIV, three colovaginoplasties and one PIV + graft) also underwent vulvar cosmetic revision surgery at our clinic during the same time period, resulting in 84 transfeminine patients that underwent cosmetic revision surgery at our centre following vaginoplasty of any provenance during the 8-year and 4-month-long study period. 8% of patients were lost in total during follow-up. Only 15.47% (13/84 transfeminine patients) that underwent cosmetic revision surgeries following vaginoplasty lived in our local community area (Catalonia; that is, in or near Barcelona) and 21.42% (18/84 patients) were smokers. Mean patient age was 36 (20–60 years old). From all performed aesthetic revision surgeries, most of them (32.38%) were labia corrections, followed by clitoris (23.07%) repair surgeries. Mons Venus (10.20%) and urethral meatus (9.38%) corrections, resection of spongiosus tissue remnants (8.57%) in the vulvar vestibule, and introitus (6.53%) repair surgeries followed in frequency. Other minor and less performed cosmetic revision surgeries involved scars satellite to the inguinal folds (5.30%) and corrections of the anterior commissure (2.84%) and inferior fourchette (2.42%) regions. No differences on topography of all cosmetic revision surgeries were found regarding vaginoplasty type (Table 1).

**Table 1:** Differences on cosmetic revision surgery (topography) among vaginoplasty types (following vaginoplasty of any provenance)

Cosmetic revision surgery	Vaginoplasty type		Difference
Topography	PIV all^a^ (*n*^b^ = 70)	Colovaginoplasty (*n* = 14)	p*
Clitoris	40	6	0.3269
Labia Majora and Minora	41	9	0.0620
Inguinal Scars	11	2	0.6893
Venus Mons	19	5	0.3128
Anterior Commissure and Inferior Fourchette	11	0	0.2256
Urethral meatus	22	1	0.3309
Introitus	10	3	0.2439
Spongiosus tissue remnants	20	0	0.0872

*p calculated with Chi2Test for variables ≥ 5 and with Fisher Test for variables < 5

^a^"PIV all" includes all inversion vaginoplasties; that is, PIV and PIV + graft

^b^*n* refers to the number of patients with vaginoplasty of any provenance that underwent cosmetic revision surgery at our institution (*n* = 84 total)

From all 84 patients who underwent vulvar cosmetic revision surgery at our clinic after transfeminine vaginoplasty, 34.52% (29/84) had more than one vulvar cosmetic revision surgery during the long-term study period (with a mean of 1.42 cosmetic revision surgeries, 3 max–1 min, STD 0.64), and in most patients, more than one vulvar topography (with a mean of 2.94 topographies per cosmetic revision surgery) was corrected in the same revision surgery. All in all, 120 vulvar cosmetic revision surgeries were analysed. The mean time period between vaginoplasty and first cosmetic revision surgery was more than 1 year (12 months with IQR 8–14 among patients with vaginoplasty performed at our centre vs 30 months with IQR 16–37 in patients who underwent vaginoplasty at other centres). 28.57% (24/84 patients) had concomitant surgical procedures with the first vulvar cosmetic revision surgery, being mainly gender confirmation surgeries involving the face (41.66%, 10/24 patients) and breast (20.83%, 5/24 patients) regions. 5.95% patients (5/84) had concomitant surgical procedures with the second or third vulvar cosmetic surgery, being breast and body contour gender-affirming surgeries the more frequent ones.

Among the patients who were found to have undergone more than one vulvar revision cosmetic surgery following vaginoplasty, no differences were encountered when analysing vaginoplasty type (*p* = 0.09) nor provenance (*p* = 0.3). Labiaplasty (35.48%) followed by clitoroplasty (20.16%) were also the two more frequent vulvar cosmetic revision surgeries after vaginoplasty in these patients, in contrast with anterior labial commissure/inferior fourchette and bulbous spongiosus remnants correction surgeries, which were the less performed (4.83% rate each).

The rate of cosmetic revision surgeries of the 354 patients who underwent transfeminine vaginoplasty at our centre during the 8-year and 4-month study period was 11.29%. Most of them were also labia (41.17%) and clitoris (19.60%) corrections. Mons Venus plasties (9.80%) and revision surgeries of scars satellite to the inguinal folds (8.82%) followed in frequency. Introitus (7.84%) and urethral meatus (6.86%) revision surgeries were also found in minor rates. Other less performed cosmetic revision surgeries involved anterior labial commissure (2.97%) and inferior fourchette (2.97%) topographic areas. In our institutional vaginoplasty cohort, no cosmetic revision surgeries regarding bulbous spongiosus and body remnants in the vulvar vestibule were found (Table 2). In this cohort of patients that underwent cosmetic revision surgery following vaginoplasty performed at our centre, no differences on cosmetic revision surgery prevalence (*p* = 0.33) (Table 3) or topography (Table 4) were seen depending on vaginoplasty type.

**Table 2:** Differences on cosmetic revision surgery (topography) (regarding vaginoplasty provenance)

Cosmetic revision surgery	Vaginoplasty provenance^a^		Difference
Topography	Our centre (*n* = 40)	Other centres (*n* = 44)	p*
Clitoris	18	28	0.865
Labia Majora and Minora	27	23	0.1556
Inguinal Scars	9	4	0.1313
Venus Mons	10	14	0.4896
Anterior Commissure and Inferior Fourchette	5	6	0.8774
Urethral meatus	7	16	0.0528
Introitus	7	6	0.6248
Spongiosus tissue remnants	0	20	**< 0.00001**

*p calculated with Chi2Test for variables ≥ 5 and with Fisher Test for variables < 5

^a^From a total of 84 transfeminine patients that underwent cosmetic revision surgery at our institution following vaginoplasty performed at our (*n* = 40) or other centres (*n* = 44)

**Table 3:** Differences on cosmetic revision surgery prevalence among vaginoplasty types (following vaginoplasty performed at our centre)

Cosmetic revision surgery	Vaginoplasty type		Difference
*n* = 354 (our centre-Vaginoplasty patients)	PIV all (*n* = 232)	Colovaginoplasty (*n* = 122)	p*
Yes (*n* = 40)	29	11	0.3251
No (*n* = 314)	203	111	

^a^“PIV all” includes all inversion vaginoplasties; that is, PIV and PIV + graft

*p calculated with Chi2Test for variables ≥ 5 and with Fisher Test for variables < 5

**Table 4:** Differences on cosmetic revision surgery (topography) among vaginoplasty types (following vaginoplasty performed at our centre)

Cosmetic revision surgery	Vaginoplasty type		Difference
Topography	PIV all^a^ (*n*^b^ = 78)	Colovaginoplasty (*n *= 24)	p*
Clitoris	17	3	0.3158
Labia Majora and Minora	30	12	0.3151
Inguinal Scars	7	2	1
Venus Mons	7	3	0.6962
Anterior Commissure and Inferior Fourchette	6	0	0.3314
Urethral meatus	7	0	0.1943
Introitus	8	4	0.4697
Spongiosus tissue remnants	0	0	–

*p calculated with Chi2Test for variables ≥ 5 and with Fisher Test for variables < 5

^a^“PIV all” includes all inversion vaginoplasties; that is, PIV and PIV + graft

^b^*n* refers to the number of patients with vaginoplasty performed at our centre that underwent cosmetic revision surgery at our institution (*n *= 84 total)

## Discussion

After transfeminine genital gender-affirming surgery, revision functional and/or cosmetic surgeries are often indicated.

In our institutional cohort of 354 patients and 8-year-long and 4-month retrospective study period, we found a 11.29% rate of cosmetic revision surgeries after transfeminine vaginoplasty performed at our centre. This is in consonance with another retrospective 10-year-long study of 189 PIV patients, where a 3–20% rate was observed [6]. They reported labiaplasty as the most common procedure performed as cosmetic revision surgery after vaginoplasty, followed by clitoroplasty. Our results also found these two surgeries, labiaplasty and clitoral repair, as the most prevalent in this population. Although our study was shorter, we included a larger number of patients, and we studied not only PIV, but also PIV + graft and colovaginoplasty patients. Up to our knowledge, this is the first study to include transfeminine patients with different vaginoplasty techniques, when analysing revision cosmetic surgeries after vaginoplasty. In this regard, we found no differences among the different studied transfeminine vaginoplasty types (inversion penile techniques versus colovaginoplasty) and cosmetic revision surgery prevalence or topography.

Other similar retrospective studies found 3–31% rates of aesthetic corrective surgeries after vaginoplasty [7–17: see Table 5 for a brief references review]. A 2-year-long study of 161 PIV patients by Opsomer et al. [14] revealed a 21.7% rate of corrective surgeries, being also the labiaplasty the most prevalent performed procedure. More in detail, it was a minor surgery and usually patient-driven. Another paper describes similar outcomes (24% rate of cosmetic revision surgeries) with 117 transfeminine patients after PIV, consisting approximately on labiaplasties or clitoroplasties, and with 8% of the cases consisting on combining revision surgeries of both regions (labia and clitoris) [9]. We also found combining revision surgeries of many vulvar topographies in the same cosmetic revision surgery procedure following transfeminine vaginoplasty. In fact, our results may suggest that lipofilling of labia majora following vaginoplasty is a common procedure that often requires more than one cosmetic revision surgery in the long term and might point out also to a higher trend of labia majora lifting procedures to achieve more aesthetic and long-lasting pleasant outcomes. Further specific studies are needed to unravel that hypothesis and further elucidate the major role that labia majora may play on transfeminine vulvar cosmesis.

**Table 5:** Literature review of cosmetic revision surgery following transfeminine vaginoplasty (main references)

Author, Location, Journal and year of publication	Study type	*n*	Vaginoplasty technique	Study period	% cosmetic revision surgery	Topography details
Opsomer et al. [14] Belgium PRS 2018	Retrospective	161	PIV	2014–2016	21.7	Labia
Boas et al. [9] US PRS 2019	Retrospective	117	PIV	2014–2016	23.8	Labia or clitoris (7.7% both)
Cristofari et al [6] France Ann Chir Plast Esthet 2018	Retrospective	189	PIV	2006–2016	3–20	Labia (20%) Clitoris (9.5%) Commissures (6.3%) Spongiosus Body (3%)
Raigosa [12] Spain J Sex Med 2015	Retrospective	60	PIV	2008–2013	21.6	Clitoris Scars Labia > Spongiosus body
Van der Sluis et al. [13] The Netherlands J Sex Med 2016	Retrospective	24	Secondary Intestinal vaginoplasty (23 sigmoid, 1 ileal)	1970–2000	NR	Labia (25%) Urethral meatus (13%)
Kaushik et al. [10] India PRS Global Open 2019	Retrospective	386	Primary sigmoid vaginoplasty	2007–2017	8.8	NR
Van der Sluis et al. [11] The Netherlands (European Multicentre Study) BJUI 2018	Retrospective	32 (27 trans)	Ileal vaginoplasty (most secondary)	NA	9.3	Labia (all)
Amend et al. [15] Germany European Urology 2013	Retrospective	24	PIV	2007–2011	54	NR
Goddard et al [16] UK BJU International 2007	Retrospective	222	PIV	1994–2004	2.5	Labia (all)
Gaither et al. [5] US JUrology 2018	Retrospective	330	Primary PIV	2011–2015	5.6	Labia (all)
Buncamper et al. [17] The Netherlands PRS 2016	Retrospective	475	PIV (405) and PIV + graft (70)	2000–2014	33.7	Labia (mainly)

As limitations of this study, the trigger of the cosmetic revision surgery indication was not studied in detail (that is, if it was more patient- or surgeon-driven). However, analysing it may also imply an added bias, as in this study examined transfeminine patients after vaginoplasty included both—operated of vaginoplasty at our centre, and also at other centres—with clear indication of aesthetic revision surgery especially in this latter case, from both a patient and surgeon perspective, as most of them were complex cases derived from other clinics due to a previous unpleasant aesthetic result. However, this bias limitation becomes also a relative advantage, as it allows us to analyse outcomes from different surgeons and show the current experience of cosmetic revision surgery following transfeminine vaginoplasty at an expert gender surgery and plastic, reconstructive and aesthetic institution. In this regard, we found that labia and clitoroplasties appeared to be the most prevalent cosmetic revision surgery topographies after transfeminine vaginoplasty in general, independently of vaginoplasty provenance and vaginoplasty technique type. However, under our study conditions, spongiosus tissue remnants clearly, followed by labia, inguinal fold satellite scars, urethral meatus and clitoris revision surgery prevalence might have a differential trend, but with our current data no conclusive statements regarding “vaginoplasty provenance” from other centres (and cosmetic revision surgery prevalence) can be done. All in all, the main strong point of this detailed descriptive study is to clearly present our vaginoplasty institution cohort data and our current state-of-the-art techniques on cosmetic revision surgeries after transfeminine vaginoplasty, which is based on more than 20 years of gender-affirming and plastic, aesthetic and reconstructive surgery experience. On the other hand, there is additional important information provided by the fact that many cosmetic revision surgeries come from vaginoplasties performed in other centres, which are usually complex surgical cases, which offers the opportunity to enrich our surgical techniques algorithm hereby shown.

Feminine genital plastic surgery in cis-females is experiencing a rapidly and increasing recognition and demand [3]. Based on our results, this appears to be the same for transfeminine patients. Providing education on female normal anatomy, real expectations and aesthetic outcomes may be helpful to better address cosmetic revision surgery following vaginoplasty, especially in the current context of anti-ageing culture and virtual network experiences such as digital pornography, online dating, social media and telemedicine, whose influence on vulvar cosmetic revision surgeries would be of great research interest, but it is outside the scope of this study.

Based on our findings, with labiaplasty followed by clitoroplasty as main cosmetic revision surgeries after transfeminine vaginoplasty, it could be hypothesized that taking special care with labia and clitoris recreation during the primary vaginoplasty procedure might be useful to decrease further cosmetic revision procedures in the near future; however, more research is required to shed more light on this issue.

## Conclusion

Cosmetic revision surgeries after transfeminine vaginoplasty are frequent. In our large and long-term cohort study, labiaplasty followed by clitoroplasty were found as the most required aesthetic revision surgical procedures. No differences regarding vaginoplasty type and cosmetic revision surgery following vaginoplasty were found. Our surgical approach is described. Further multicentre, prospective and controlled studies are necessary to improve aesthetic outcomes and scientific evidence after transfeminine vaginoplasty.
